# Intervention of computer-assisted cognitive training combined with occupational therapy in people with mild cognitive impairment: a randomized controlled trial

**DOI:** 10.3389/fnagi.2024.1384318

**Published:** 2024-05-10

**Authors:** Xin Wen, Shangrong Song, Hui Tian, Hang Cui, Lijuan Zhang, Yajie Sun, Mingyue Li, Yonghong Wang

**Affiliations:** ^1^The First Hospital of Jilin University, Changchun, Jilin, China; ^2^School of Nursing, Jilin University, Changchun, Jilin, China

**Keywords:** mild cognitive impairment, computerized cognitive training, occupational therapy, cognitive function, anxiety, depression

## Abstract

**Objective:**

Investigate the impact of combined computerized cognitive training and occupational therapy on individuals with mild cognitive impairment (MCI).

**Methods:**

We randomly assigned 118 MCI patients into two groups: a combined intervention group (*n* = 37) and a control group (*n* = 81), the latter receiving standard nursing care. The intervention group additionally underwent 12 weeks of computerized cognitive training and occupational therapy. Blind assessors evaluated cognitive performance, anxiety, depression, and daily living activities before the intervention, post-intervention, and at a 3-month follow-up.

**Results:**

Repeated-measures analysis of variance showed that the sMoCA scores, HAMA scores, and ADL scores of the experimental group at T2 (post-intervention) and T3 (3-month follow-up) were higher than those of the control group, and the difference was statistically significant (*p* < 0.001, *p* < 0.001, *p* = 0.026).

**Conclusion:**

Computerized cognitive training combined with occupational therapy can improve patients’ cognitive status, enhance their compliance with continuing care, and maintain their anxiety and self-care ability at a stable level.

**Clinical trial registration:**

https://www.chictr.org.cn/index.html, identifier ChiCTR2200065014.

## 1 Introduction

The global population’s aging is leading to an increase in dementia prevalence, posing a significant challenge to worldwide health and social care systems in the 21st century (Chowdhary et al., 2021). Mild cognitive impairment (MCI) represents a transitional stage from the normal cognitive decline associated with aging to dementia-induced cognitive impairment (Li et al., 2022; Laffont et al., 2020; Gomez-Soria et al., 2022). A recent study found that 6.0% of Chinese over 60 have dementia, totaling approximately 15.07 million people, while MCI affects 15.5% or 38.77 million (Jia et al., 2020). Dementia can lead to progressive cognitive deficits, dysfunction, and behavioral changes in patients, which lead to stigma and social isolation and impose heavy social and economic burdens (Shah et al., 2016; De la Rosa et al., 2020; Heins et al., 2021). Besides cognitive decline, MCI patients often exhibit psycho-behavioral symptoms like anxiety and depression, among the most critical neuropsychiatric symptoms (Ma, 2020). Thus, delaying MCI’s progression is crucial to slowing patients’ cognitive decline and reducing caregivers’ burden. Early MCI interventions encompass both pharmacological and non-pharmacological treatments (Wang et al., 2023). Given the limited efficacy of drugs, non-drug therapies are now the focal point for delaying MCI progression (Ligo Teixeira et al., 2012; Cooper et al., 2013).

Early non-pharmacological interventions such as exercise, cognitive rehabilitation, and occupational therapy can slow the decline in cognitive function of people living with MCI (Spijker et al., 2008; Bahar-Fuchs et al., 2019; Clare et al., 2019). Cognitive training is acknowledged as an effective non-pharmacological method to treat cognitive impairment, attributed to its proven learning potential and cognitive plasticity (Li et al., 2011; Xue et al., 2023). Advances in computer technology have led to the replacement of traditional cognitive training with computerized cognitive training (CCT), allowing for interactive exercises via computers, tablets, VR, and other devices (Li et al., 2022). CCT offers more engaging activities, immediate feedback, and customization compared to traditional methods, enhancing ease of use and personalization (Park et al., 2020). The game-like design of CCT is believed to provide intrinsic rewards (Caggianese et al., 2018). Furthermore, CCT’s ease of use, affordability, and scalability make it a growing choice for MCI interventions to enhance cognitive functions (Liang et al., 2018).

Numerous studies indicate CCT improves cognitive function in the elderly (Lampit et al., 2015; Yu et al., 2021). Yet, its long-term sustainability remains uncertain, with scholars advocating for ongoing, continuous application (Reuter-Lorenz and Park, 2014; Sherman et al., 2017; Zhang et al., 2019). In China, CCT is primarily conducted in hospitals under cognitive trainers’ supervision, requiring patients to often have family accompany them (Chan et al., 2024). In addition, assistive technologies can help maintain the health and independence of older adults, support greater participation in meaningful community and individual activities, and help provide a better sense of security, peace of mind, and communication. However, while older people are increasingly interested in such technologies, their acceptance, adoption and availability are lower compared to younger people. Two studies found that the willingness of the elderly to use assistive technology is complex, one study showed that age, gender, etc. are important predictors of the willingness of the elderly, and another study showed that the elderly in the United States are more willing to use smart home technology, which limits the application of CCT (Kadylak and Cotten, 2020; Begde et al., 2024). Challenges in maintaining CCT’s therapeutic effect arise from time, staff, and financial constraints. Additionally, the significant yet frequently overlooked role of daily home training is crucial.

Consequently, this study combines inpatient CCT with home-based occupational therapy to address these challenges. To date, no studies in China have investigated CCT combined with OT for MCI patients. Occupational therapy (OT), as a key aspect of rehabilitation, helps those with disabilities engage in meaningful activities to prevent, restore, or mitigate dysfunction. The promoted activities include personal care, leisure and entertainment, and productive activities, facilitating the recovery of physical, psychological, and social adaptive functions (Laffont et al., 2020). CCT primarily focuses on using computer programs to improve specific cognitive functions such as memory, attention, and so on. OT focuses on helping patients recover or improve their functional abilities in daily life and work, including physical, emotional, and cognitive levels. The complementarity of CCT and OT is reflected in that CCT strengthens specific cognitive functions through targeted technical means, while OT helps patients apply these improved cognitive abilities to daily life and social activities to enhance self-care ability and social participation. By combining the two, the improvement of cognitive function and its effective application in real life can be achieved, thus more comprehensively supporting the rehabilitation of patients and improving the quality of life. This study is based on the principles of cognitive stimulation and cognitive training as well as an operational model (Chunshan et al., 2018; Clare et al., 2023), finally developed a joint intervention plan implemented by rehabilitation therapists based on in-hospital computer cognitive training and out-of-hospital OT. The rehabilitation therapists in this study played the role of implementing CCT, OT treatment and making rehabilitation plans. Based on the results of the evaluation, the rehabilitation therapist develops an individualized treatment plan aimed at helping the patient regain normal physical function or improve existing function. The purpose of this study was to investigate the effects of CCT combined with OT on cognitive function, anxiety, depression and activities of daily living in patients with MCI in the context of classic paper-and-pencil cognitive training. The core hypothesis is that CCT combined with OT improves MCI patients’ cognitive function. The secondary hypothesis suggests CCT and OT together can also enhance daily living functions and reduce anxiety and depression in MCI patients.

## 2 Methods and participants

### 2.1 Study design

The study was registered with the Chinese Clinical Trial Registry (Trial I.D.: ChiCTR2200065014) and followed the Consort Group Guidelines (Supplementary material) (Moher et al., 2010). In an assessor-blind randomized controlled trial conducted in Changchun, Jilin Province, China, we randomly assigned subjects to experimental and control groups (assignment ratio 1:2). The trial group received the intervention immediately after randomization, while participants assigned to the control group were offered the experimental intervention after the experiment ended. The assessment was performed at three time points: pre-intervention (at baseline) T1, immediately after intervention T2, and 3 months after intervention (at follow-up) T3.

The 1:2 assignment of the trial group to the control group was chosen because the experiment involved a new, as-yet-unestablished intervention and was designed to help improve the statistical power of the study in order to gain experience in implementing the intervention. In the given parameter Settings, the statistical power of 1:1 matching is about 27.5%, and the statistical power of 1:2 matching is about 34.9%. However, compared with 1:1 matching, 1:2 matching can provide more control data and enhance statistical power, but it also has its disadvantages and potential impacts: (1) Resource constraints: Conducting 1:2 matching may require more time and resources to identify and collect suitable control members; (2) Data consistency issues: Increasing the number of control samples may introduce more variability, especially if the members of the control group are not sufficiently similar on important covariates. This suggests that increasing the proportion of the control group from 1:1 to 1:2 can indeed increase the statistical power of the study, although the increase in effect is not very significant, but in some cases this increase may be crucial to detect smaller effects. The improved efficacy in this case reflects the advantage of increasing the size of the control sample.

### 2.2 Research environment and participants

From October 2020 to June 2022, 118 MCI patients admitted to the memory clinic of a grade-A hospital in Changchun were recruited to participate in this study. The inclusion criteria were as follows: (1) aged 45 years or older, (2) met the diagnostic criteria for MCI of the 2018 Chinese Guidelines for the Diagnosis and Treatment of Dementia and Cognitive Impairment Diagnosis and Treatment of Mild Cognitive Impairment (Jia Jian-ping and Cui-bai, 2011), (3) able to cooperate with the daily inspection and had adequate levels of language expression and comprehension, and (4) not participating in standardized and systematic cognitive training and cognitive stimulation programs in the past year or during the same period, and agreed to take part in this study, (5) all patients lived in urban communities and had the financial ability to complete one course of cognitive training. The exclusion criteria were as follows: (1) unable to carry out activities of daily living (ADL ≤40), or (2) physical severe diseases, mental disorders (for example, HAMA score for anxiety ≥14 or HAMD score for depression ≥17), or long-term use of psychotropic drugs that may affect cognitive function. The intervention participants were professional rehabilitation nurses with rich experience in the field and authoritative certification.

The sample size was determined using G*Power analysis, a widely accepted method for power analysis in clinical trials (Faul et al., 2007). Previous studies, such as those by Gomez-Soria et al. (2020) and Tao et al. (2023), have utilized similar parameters for effect size and power to establish robust sample sizes in clinical trials involving cognitive interventions. In our study, with a planned statistical power of 0.9 and an effect size (d) of 0.6, it was calculated that a minimum of 102 individuals (experimental group = 34, control group = 68) would be needed to ensure sufficient power, with a 95% confidence interval and a 5% type two error.

### 2.3 Randomization and blind method

Nursing staff at participating hospitals identified potential participants from their medical records and sent invitations to potential participants. The principal investigator (PI) assessed the patients’ eligibility and sought their written informed consent. Eligible subjects were randomly assigned to the experimental or wait-list control groups. Randomization was performed by researchers not affiliated with the current study using the random number computing program^1^. It was not feasible to blind subjects and interveners, but assessors for both groups were blinded to participants’ assigned conditions.

### 2.4 Intervention

Based on a literature review, the first draft of the joint intervention program was developed. Eight experts in neurology, psychiatry, rehabilitation, and nursing were consulted to provide feedback on the planned intervention program. Before the formal implementation of this intervention, four patients were selected to conduct pilot testing, and no adverse reactions were observed. The intervention process is shown in Table 1.

**Table 1:** Intervention process.

Intervention	Intervention time	Intervention content
Routine care	For 12 weeks	classic paper-and-pencil cognitive training: ① The nurse led the patient to read some simple and easy to understand books, and let the patient talk about their understanding; ② The nursing staff provided the patient with a paper with 5 numbers, guided the patient to memorize for 2 min, and then the patient recorded it on the paper. The nursing staff carried out disease health education to the patients or caregivers, follow-up visits were made three times a week, and family members were instructed to monitor their cognitive symptoms and timely detection of adverse conditions of patients. Instruct family members to care for and encourage the patient and to follow specific MIND (Mediterran-DASH Intervention for Neurodegenerative Delay) diet and aerobic exercise guidelines. MCI health lectures were held regularly to provide patients and their families with continuous nursing services in the form of consultation, telephone follow-ups, and network exchanges.
In-hospital CCT	For 12 weeks, three sessions per week, with sessions ≥30 min in length	After the baseline characteristics of patients were assessed with the BH6 brain desktop application software developed by Beijing Zhizhi Technology Co., Ltd., various tasks personalized in terms of difficulty were administered. Each session involved three stages: In the first stage of training (5 min), the adaptation stage, the patient selects simple training, such as finger operation and training or addition and subtraction. The second stage was formal training (25–30 min). Training consisted of 4–5 training tasks (for attention, processing speed, long-term memory, working memory, flexibility, sensory perception, or other cognitive domains). The third stage of training was relaxation (5–10 min); this stage involved independent training on previous areas to enhance performance in weaker areas from the second stage or meditation relaxation with music therapy. The computer automatically adjusts the difficulty of the training task according to the individual’s performance. After the individual completed each training task, they were rewarded with videos or pictures.
Out-of-hospital, at-home OT	For 12 weeks, maintain three tasks four times a week, with no limit on training time, until the homework content is completed	Rehabilitation therapists interview family members about what the patient is familiar with and interested in. OT is carried out in various forms, such as recreational activities, games, handicrafts, diary writing, and housework, and the difficulty is gradually deepened. The family caregiver records each completion. Every Sunday, the rehabilitation therapist will contact the family through WeChat, supervise the task completion according to the record, and adjust the content of OT according to the completion to ensure the continuity of training.

#### 2.4.1 Control group

The control group received routine care, in addition to disease observation, safety management, disease knowledge, and health education, classic paper-and-pencil cognitive training (includes a variety of cognitive exercises, such as puzzles and memory tasks, designed to stimulate different cognitive functions, and systematically randomly adjusts the type and difficulty of the exercises to prevent learning bias), exercise, dietary advice, social communication and other aspects of oral health education guidance and regular follow-up. The specific contents of routine care were as follows: nursing staff issued printed health guidance materials to patients and their families, follow-up visits were made three times a week, and family members were instructed to monitor their cognitive symptoms and timely detection of adverse conditions of patients. Family members were asked to care for and encourage patients, patiently provide daily companionship and care, and follow specific MIND (Mediterranean-DASH Intervention for Neurodegenerative Delay) diet and aerobic exercise guidelines. MCI health lectures were held regularly to provide patients and their families with continuous nursing services in the form of consultation, telephone follow-ups, and network exchanges to ensure that the rehabilitation needs of patients were met. The control group was provided with the intervention measures as needed after the study was completed to protect the rights and interests of patients.

#### 2.4.2 Experimental group

The experimental group received 12 weeks of CCT combined with cognitive OT and the care provided to the control group. The specific details of the intervention are as follows:

1. In-hospital computer-assisted cognitive training: CCT was administered for 12 weeks, three sessions per week, with sessions ≥30 min in length. Each session involved three stages. In the first stage of training (5 min), the adaptation stage, the patient selects simple training, such as finger operation and training or addition and subtraction. The second stage was formal training (25–30 min). After the baseline characteristics of patients were assessed with the BH6 brain desktop application software developed by Beijing Zhizhi Technology Co., Ltd., various tasks personalized in terms of difficulty were administered. Participants completed two training rounds within 25–30 min, each with 4–5 training tasks. Each training task targeted attention, processing speed, long-term memory, working memory, flexibility, sensory perception, or other cognitive domains. The computer automatically adjusts the difficulty of the training task according to the individual’s performance. After the individual completed each training task, they were rewarded with videos or pictures. The third stage of training was relaxation (5–10 min); this stage involved independent training on previous areas to enhance performance in weaker areas from the second stage or meditation relaxation with music therapy. During the CCT, the trainer coaches the participants one-on-one based on their cognitive assessment performance in each class.
2. Out-of-hospital, at-home occupational therapy: The rehabilitation therapists who provided cognitive training individually implemented multi-field OT for the people with MCI, maintaining three tasks each time four times a week, with no limit on the training time, to complete the homework content. OT selects the content that the patient is familiar with and interested in and carries out in various forms, such as recreational activities, games, handicrafts, diary writing, and housework, and the difficulty is gradually deepened. The family caregiver is asked to keep a record of each completion. Every Sunday, the rehabilitation therapist will contact the family through WeChat, supervise the task completion according to the record, and adjust the content of OT according to the completion to ensure the continuity of training.

#### 2.4.3 Additional procedures for interventions

Before the main intervention, staff led a one-day course on the trial procedures. Staff were trained in safe and ethical treatment practices as per standardized procedures. Concurrently, the team provided materials and rules, while experimenters recorded and scored results based on these materials. Participants were informed they could discontinue treatment anytime. Simultaneously, the team monitored program impacts to prevent adverse events. A pre-intervention session ensured participants could adhere to the trial protocol. Staff checked for and reported adverse events before and during each intervention. These covered any physical or mental discomfort, stopping the trial for participant withdrawal and readmission, or observing significant mental state changes.

### 2.5 Evaluation tools

The Mini-Mental State Examination and the Montreal Cognitive Assessment Scale were used to determine cognitive function, the Hamilton Anxiety Scale and the Hamilton Depression Scale were used to assess emotional state, and the basic activities of daily living and instrumental activities of daily living were considered with the Instrumental Activities of Daily Living (IADL) scale.

1. The Mini-Mental State Examination (MMSE) was created by Folstein et al. (1975). A total of 30 items are used to assess five dimensions: attention, memory, cognitive function, orientation and numeracy, language ability, and recall ability. The total score ranges from 0 to 30; higher scores are associated with better cognitive function. Cronbach’s α coefficient was 0.950, and the test–retest reliability was 0.900.
2. The Montreal Cognitive Assessment Scale (MoCA), developed by Professor Nasreddine in 2004 (Nasreddine et al., 2005), is a unique assessment tool used to screen for MCI rapidly. It includes 12 items, with a total score of 30. Scores ≥26 indicate normal cognitive function; the total score was increased by 1 point if the patient has less than 12 years of education.
3. The Hamilton Anxiety Scale (HAMA) and Hamilton Depression Scale (HAMD) were compiled by Hamilton (1960), respectively. With good reliability and validity, these scales have been widely used in psychiatric clinical assessment. They can be used to evaluate the therapeutic effect on mental disorders and compare symptoms before and after treatment. On the HAMA, scores of 0 ~ 7 indicate no anxiety, scores of 8 ~ 14 indicate suspected anxiety, scores of 15 ~ 21 indicate mild anxiety, scores of 22 ~ 29 indicate moderate anxiety, and scores ≥30 indicate severe anxiety. On the HAMD, a score of 7 indicates no depression, a score of 7 ~ 17 indicates mild depression, a score of 17 ~ 24 indicates moderate depression and a score of 24 indicates severe depression.
4. Activities of daily living (ADL), a concept proposed by Sidney Katz in 1963, refer to the necessary activities a person performs daily to meet their daily needs. These activities reflect the most fundamental capacities of people in the home, health care facility, or community. The IADL scale includes basic activities (basic ADL; 8 items) (Katz and Akpom, 1976) and instrumental activities (12 items) (Rosow and Breslau, 1966). A score of 1 is classified as usual, 2–4 is classified as functional decline, scores on two or more items ≥3 indicate obvious functional impairment, and a total score ≤ 26 is classified as usual. The Cronbach’s alpha values of ADL and IADL were 0.879 and 0.802 respectively, both of which had good reliability (Dong et al., 2014; Zhao et al., 2022).
5. Compliance: Nurses recorded the number of cognitive training and occupational therapy sessions completed by patients weekly. Good compliance was defined as ≥3 sessions of cognitive training and homework conducted per week, while poor compliance was defined as ≤2 sessions. Weekly statistics and feedback on compliance were delivered.

### 2.6 Data collection

Before the implementation of the intervention, strict training and division of labor were conducted for the group members. To reduce bias in data collection, the same cognitive therapist (blinded to group allocation) assessed the indicators of both groups before and after the intervention; a different experimenter conducted cognitive training for the patients. Data were collected at baseline (T1), after 12 weeks of the intervention (T2), and 3 months after the intervention (T3) had been completed (follow-up visit). The primary outcome measure of this study was cognitive performance. Secondary outcome measures were anxiety, depression, and ability to perform activities of daily living. The researcher explained other items in the questionnaire to the patients using standardized guidance. After the patients answered, the researcher completed the questionnaire, and other group members completed the data verification and entry. In addition, trial funders are not involved in data collection, interpretation, or publication of results throughout the trial.

### 2.7 Statistical method

Epidata3.0 was used to establish a database, and IBM SPSS 24.0 was used to calculate general descriptive statistics and group comparisons. Normally distributed data are described by the mean ± standard deviation, and the test was used for group comparisons. The frequency and percentage represent Categorical data, and group comparisons involved χ^2^ or Fisher exact tests (when the minimum expected value was <5). Repeated-measures ANOVAs were used for comparisons of multiple variables. The significance level (α) was set at 0.05. All the participants were included in the intention-to-treat (ITT) analysis.

### 2.8 Ethical approval

This study has been approved by the Ethics Committee of the Nursing School of Jilin University (approval number: 2022062201) and implemented by the Declaration of Helsinki. All participants were fully informed about the voluntary nature of their participation and their right to withdraw from the study at any time. Data collection takes place only with the participants’ written informed consent, and all study data is held using a secure and encrypted platform.

## 3 Results

### 3.1 Comparison of general data between the two groups

**Figure 1:**
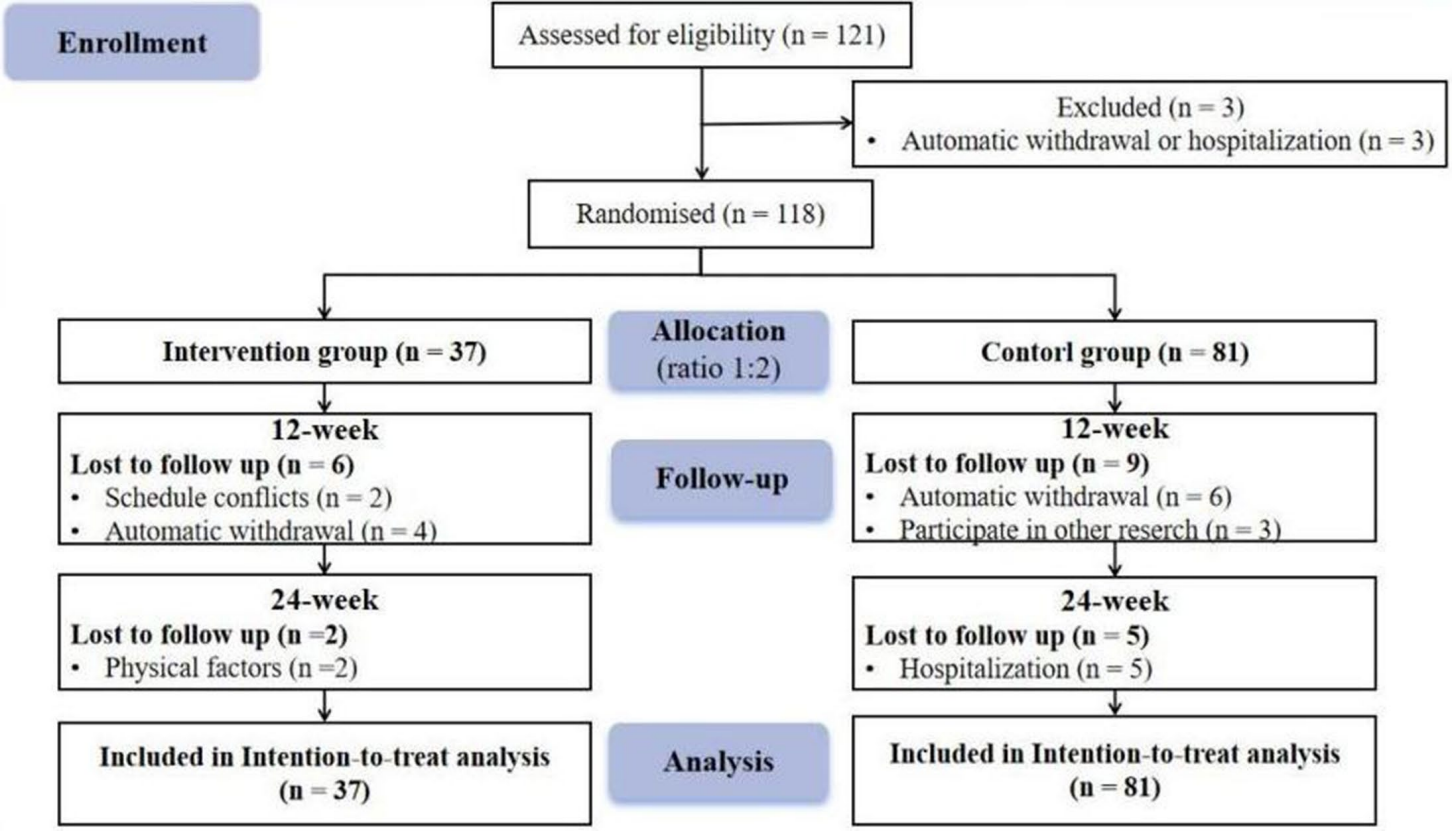
Study flowchart.

One hundred twenty-one people were recruited, 3 of whom were subsequently excluded due to automatic withdrawal or hospitalization. One hundred eighteen patients completed the study. The participants were initially allocated to the experimental group and control group at a ratio of 1:2. There were 37 individuals in the experimental group and 81 individuals in the control group. Because some patients were missing due to some reasons, we used ITT analysis to ensure the integrity of the data. This method can improve the internal validity of the study, reduce Class I errors, make the results more conservative, and maintain statistical power (Andrade, 2022). In addition, for missing data, we use “last-observation-carried forward” (LOCF) to solve (Lachin, 2016). The participant flow chart is shown in Figure 1. Before the intervention, the control and experimental groups did not differ in any variables except for stroke history (*p* > 0.05); intervention is possible, as shown in Table 1.

### 3.2 Compliance results

The study reports that after the 12-week intervention, 83.78% of the patients had good compliance, while 16.22% had poor compliance. In the experimental group, 94.59% of the patients had good compliance with follow-up, whereas only 5.40% had poor compliance due to physical factors. These findings indicate that the combination of cognitive training and occupational therapy is a highly effective intervention for people with MCI and is well-tolerated by most patients.

### 3.3 Outcome measures

**Figure 2:**
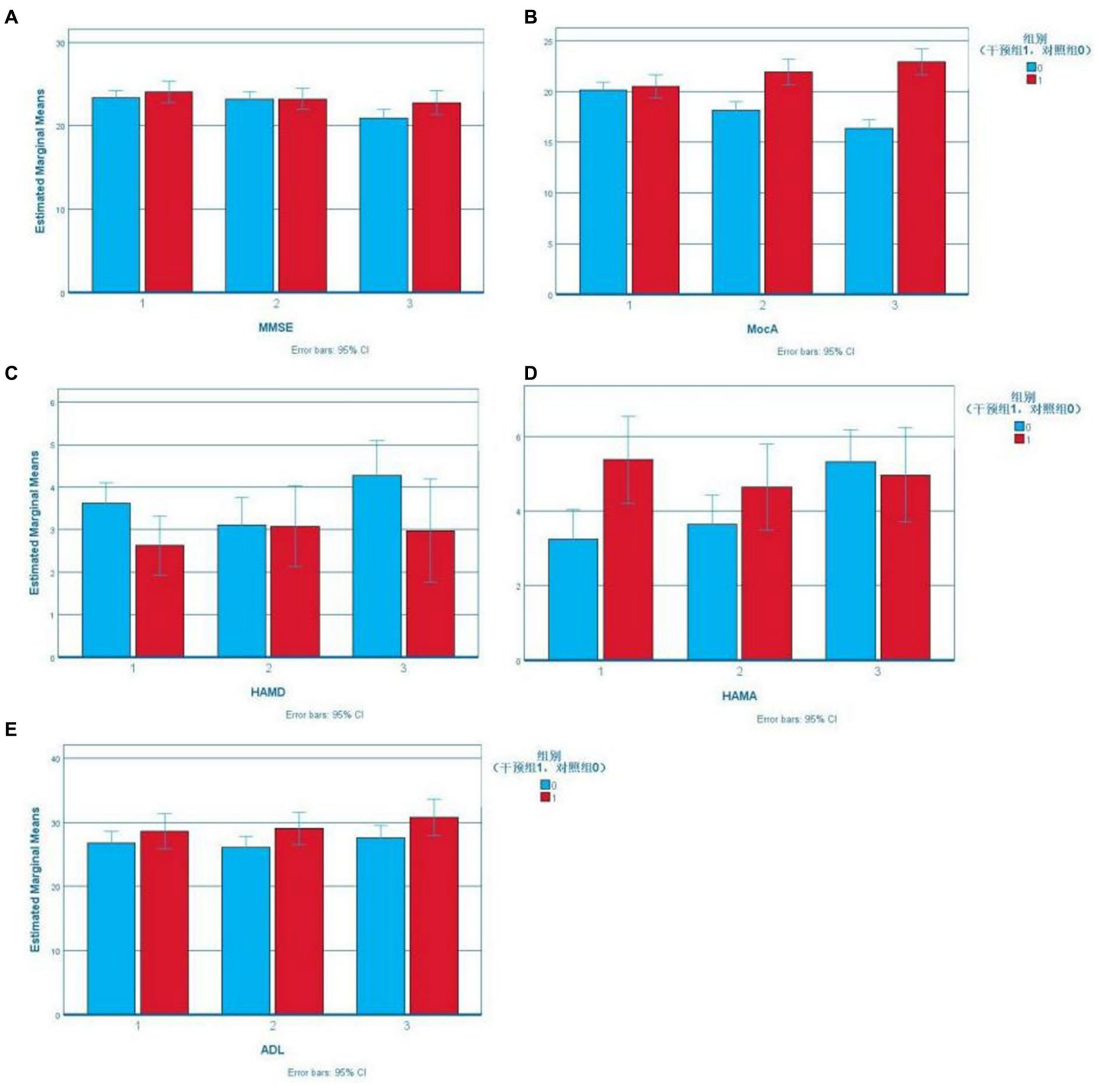
Error bar chart.

According to boxplots, the data were not normally distributed. Stroke history was included as a covariable to determine the effect of the intervention on the cognition, emotion, and daily life activities of the study subjects, as shown in Table 2. The Error Bar Chart of each scale is shown in Figure 2.

**Table 2:** Comparison of basic data between the two groups.

Category		Control group (*n* = 81)	Experimental group (*n* = 37)	*t/x* ^*2*^	*p*
Age		65.75 ± 9.405	66.78 ± 8.300	−0.572	0.568
Sex	Male	49 (60.5%)	21 (56.8%)	0.147	0.840
	Female	32 (39.5%)	16 (43.2%)		
Educational level	Primary and below	6 (7.4%)	1 (2.7%)	1.008	0.614
	Junior and senior high schools	49 (60.5%)	22 (59.5%)		
	University and above	26 (32.1%)	14 (37.8%)		
Occupation	Physical strength is dominant	35 (43.2%)	13 (35.1%)	0.686	0.428
	Brain-based	46 (56.8%)	24 (64.9%)		
Matrimony	Married	78 (96.3%)	37 (100.0%)	——	0.551
	Other	3 (3.7%)	0 (0.0%)		
Handedness	Dextromanuality	64 (79.0%)	35 (94.6%)	4.565	0.056
	Left-handedness	17 (21.0%)	2 (5.4%)		
Gait change	No	78 (96.3%)	37 (100.0%)	——	0.551
	Yes	3 (3.7%)	0 (0.0%)		
History of stroke	No	59 (72.8%)	19 (51.4%)	5.234	0.035
	Yes	22 (27.2%)	18 (48.6%)		
History of head trauma	No	79 (97.5%)	36 (97.3%)	——	1.000
	Yes	2 (2.5%)	1 (2.7%)		
Hypertension	No	48 (59.3%)	16 (43.2%)	2.625	0.116
	Yes	33 (40.7%)	21 (56.8%)		
hypercholesterolemia	No	72 (88.9%)	36 (97.3%)	——	0.168
	Yes	9 (11.1%)	1 (2.7%)		
Diabetes	No	61 (75.3%)	26 (70.3%)	0.333	0.653
	Yes	20 (24.7%)	11 (29.7%)		
Heart disease	No	64 (79.0%)	25 (67.6%)	1.795	0.249
	Yes	17 (21.0%)	12 (32.4%)		
Family history of dementia	No	77 (95.1%)	37 (100.0%)	——	0.307
	Yes	4 (4.9%)	0 (0.0%)		
Smoking history	No	57 (70.4%)	28 (75.7%)	0.355	0.660
	Yes	24 (29.6%)	9 (24.3%)		
Drinking history	No	63 (77.8%)	30 (81.1%)	0.166	0.810
	Yes	18 (22.2%)	7 (18.9%)		

#### 3.3.1 Effect of CCT combined with OT on primary outcome cognitive function

MMSE scores did not satisfy the assumption of equal variance according to Mauchly’s sphericity test (*p* = 0.011), and there was no statistical significance in MMSE scores between the two groups (*p* > 0.05). Time significantly affected MMSE scores (*p* < 0.001), indicating they were among the time points. Further pairwise comparison within the groups showed that the MMSE score of the control group at follow-up (20.93 ± 4.460) was lower than that before the intervention (23.33 ± 4.114), with a difference of 2.407 (95% CI: 1.507–3.308); the difference was statistically significant (*p* < 0.001).

According to Mauchly’s sphericity test, MoCA scores did not satisfy the assumption of equal variance (*p* < 0.001). The group-by-time interaction significantly affected MoCA scores (*p* < 0.001). The MoCA scores of the experimental group were higher than that of the control group (18.14 ± 4.203) after the intervention (21.95 ± 3.064) and at follow-up (22.95 ± 3.240). The difference was statistically significant (*p* < 0.001). The MoCA scores of the intervention group increased gradually over time, and the difference among the three-time points was statistically significant (preintervention vs. postintervention: *p* = 0.009, postintervention vs. follow-up: *p* = 0.017, preintervention vs. follow-up: *p* < 0.001). The MoCA scores of the control group decreased gradually, and pairwise comparisons of the three measurements showed statistically significant differences (*p* < 0.001; Figure 2).

#### 3.3.2 Effects of CCT combined with OT on secondary outcomes of anxiety and depression and activities of daily living

According to Mauchly’s sphericity test, HAMA scores satisfied the assumption of equal variance (*p* = 0.121). There was a significant main effect of time on HAMA scores (*p* < 0.001). Further pairwise comparison within groups showed that the HAMA scores of the control group at follow-up were higher than those before and after the intervention, and the difference was statistically significant (*p* < 0.001).

According to Mauchly’s sphericity test, HAMD scores did not satisfy the assumption of equal variance (*p* = 0.004). There were no significant effects of group, time, or group-by-time interaction on HAMD scores (*p >* 0.05).

**Figure 3:**
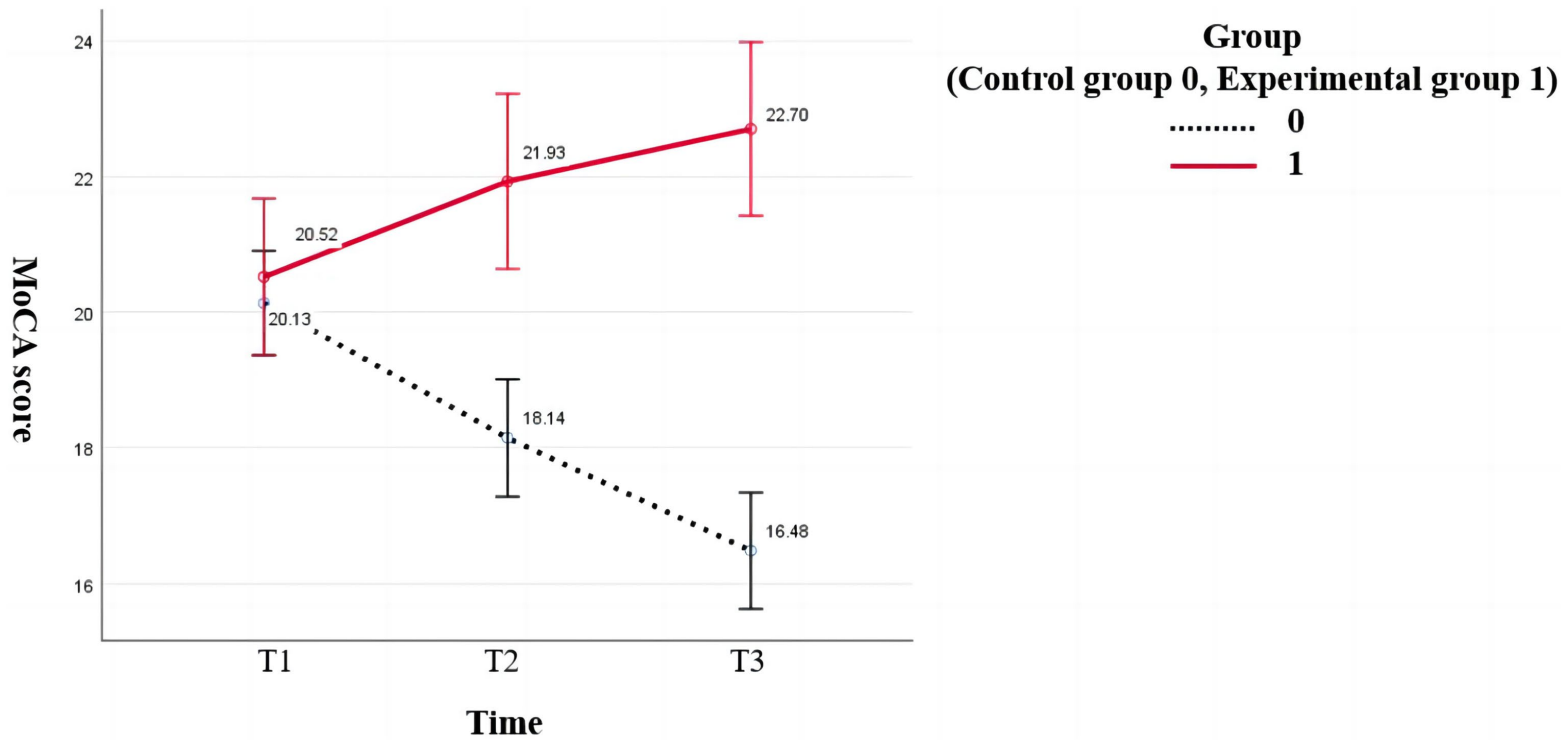
Changes in MoCA scores of the two groups at different time points.

According to Mauchly’s sphericity test, the ADL scores satisfied the assumption of equal variance (*p* = 0.365). There was a significant main effect of time on ADL scores (*p* < 0.05). Further pairwise comparison within groups showed that the ADL scores of the control group after the intervention were lower than those at follow-up, and the difference was statistically significant (*p* = 0.026) (Figure 3).

## 4 Discussion

The study used in-hospital CCT combined with out-of-hospital OT to explore the effects on cognitive function, anxiety and depression, and activities of daily living in patients with mild cognitive impairment. The results showed that CCT combined with multimodal OT significantly improved cognitive function and anxiety in patients with MCI, but had no significant effect on depression. In addition, the study also found that CCT combined with OT improved ADL in patients with MCI. Except for depression, the results were consistent with the original hypothesis.

### 4.1 Primary outcome

The main result that CCT combined with OT can significantly improve cognitive function in patients with MCI is consistent with the results of two Chinese studies on CCT and multimodal OT (Jiangbo, 2023; Yingjuan, 2023). Jiangbo et al. have pointed out that the implementation of homework therapy can improve cognitive function in patients with Alzheimer’s disease. Yingjuan et al. showed that computerized cognitive training can help improve the overall cognitive function of patients with mild cognitive impairment. In addition, this study also found that there were significant group differences in MoCA scores and no significant group differences in MMSE scores. Previous studies comparing these scales (Zhan Xiuling et al., 2021; Huifeng and Yuanrong, 2022) showed that the MoCA has better sensitivity than the MMSE for detecting MCI, which is further supported by the results of this study.

During CCT, the rehabilitation therapists guided participants to further improve performance in areas of weakness according to the cognitive assessment in each session and encouraged participants to improve verbal and nonverbal communication. The above measures led to the continuous activation of brain regions that support orientation, attention, calculation, and language ability, thereby prompting neuronal repair that would improve cognitive function (Zhan Xiuling et al., 2021). To further consolidate the benefits of in-hospital computer training, the therapists provided online supervision, and the caregivers provided offline coordination for the implementation of OT, constantly adjusting the task in response to the patient’s cognitive level and making personalized arrangements to ensure that patients with different abilities were able to participate in these activities. OT can promote patients’ visual and auditory responses, improve their attention and cognitive abilities, and enhance feelings and perceptions (Shujiao, 2018). The combination of the two methods not only improved cognitive function but also further improved patients’ compliance with independent traditional cognitive training and multimodal after the cognitive training intervention had ceased (i.e., during the follow-up period), leading to steady improvements in cognitive function that maintained patients’ quality of life and social functioning. In addition, studies have shown that extending training for as long as possible can enhance or maintain training-related cognitive benefits and provide more excellent resistance to cognitive decline (Coyle et al., 2015). The reason may be that as the disease progresses, the compensation mechanisms established during training decline, and the benefits of training slowly dissipate.

### 4.2 Secondary outcomes

In this study, we actively explored anxiety and depression in people with MCI, and the results showed that HAMA score had statistical significance between groups, while HAMD score had no significant effect on group, time and inter-group interaction, indicating that CCT combined with OT significantly improved anxiety but had no significant effect on depression. This is inconsistent with the results of prior studies. A meta-analysis showed that CCT led to substantial improvements in major depressive disorder (Motter et al., 2016). The reason for this may be that the subjects in this study did not stop the use of antidepressants, psychotherapy, or transcranial direct current stimulation, which may have interfered with the results of this study and amplified the effects of cognitive training. In addition, similar interventions or studies in similar populations have reported improvements in depression and anxiety (Xie Ping-Xiang et al., 2022; Wei et al., 2023), which may be related to the exclusion of patients with severe mental illness, and the intervention program in this study was mainly aimed at improving the cognitive function of MCI patients, without specific methods to improve mood, such as music therapy (Lam et al., 2020). In this study, only the control group exhibited increased anxiety at follow-up, which may be related to the change in the patient’s condition over time; the anxiety and depression levels of the experimental group were maintained at a stable level. Therefore, large-scale randomized controlled trials are needed in the future to validate further the effect of CCT combined with OT on depression.

CCT combined with OT led to improvement in the ADL scores of people with MCI, as the ADL scores of the intervention group were lower after intervention than at follow-up. Compared to the previously reported lack of positive effects (Loewenstein et al., 2004; Talassi et al., 2007), the results of this research show some promise, which aligns with the results of another study of combined recall therapy (Barban et al., 2016). However, there was no significant improvement in ADL scores in this study, and the results showed that the intervention group had lower ADL scores after the intervention than at follow-up, which may be because time has a significant main effect on ADL scores, and the 12-week intervention time in this study was not enough to show a significant effect on ADL. In addition, although the OT program in this study involved recreational activities and housework, it focused on cognitive aspects, such as recitation of ancient poems and diary writing, after taking into account the interests of the patients and in conjunction with the main purpose of the study. Future studies can increase the proportion of tasks involving activities of daily living, further explore intervention effects, and help people with MCI achieve a good quality of life (Table 3).

**Table 3:** Comparison of cognitive scale scores between the two groups.

Category		T1	T2	T3	ω^2^	ω^2^ 95%CI
MMSE	Control group	23.33 ± 4.114	23.15 ± 4.062	20.93 ± 4.460	0.16	0.08, 0.24
	Experimental group	24.05 ± 3.865	23.22 ± 3.831	22.78 ± 4.691		
	*F*_inter-group_/*P*	0.651/0.422				
	*F*_time_/*P*	14.037/<0.001				
	*F*_interaction_/*P*	3.037/0.052				
MOCA	Control group	20.15 ± 3.818	18.14 ± 4.203	16.37 ± 4.203	0.02	0.00, 0.06
	Experimental group	20.49 ± 2.556	21.95 ± 3.064	22.95 ± 3.240		
	*F*_inter-group_/*P*	27.841/<0.001				
	*F*_time_/*P*	6.927/0.001				
	*F*_interaction_/*P*	32.264/<0.001				
HAMA	Control group	3.05 ± 2.132	3.65 ± 3.298	5.32 ± 3.507	0.01	0.00, 0.05
	Experimental group	3.81 ± 1.853	4.65 ± 4.015	4.97 ± 4.652		
	*F*_inter-group_/*P*	0.206/0.650				
	*F*_time_/*P*	5.670/0.004				
	*F*_interaction_/*P*	2.221/0.113				
HAMD	Control group	3.63 ± 2.244	3.11 ± 2.958	4.27 ± 3.867	0.04	0.00, 0.09
	Experimental group	3.57 ± 2.577	3.08 ± 2.793	2.97 ± 3.346		
	*F*_inter-group_/*P*	1.297/0.257				
	*F*_time_/*P*	2.621/0.077				
	*F*_interaction_/*P*	1.758/0.177				
ADL	Control group	26.80 ± 7.840	26.10 ± 7.224	27.62 ± 7.615	0.04	0.00, 0.09
	Experimental group	28.62 ± 9.552	29.05 ± 9.107	30.73 ± 10.994		
	*F*_inter-group_/*P*	2.419/0.123				
	*F*_time_/*P*	4.349/0.015				
	*F*_interaction_/*P*	0.990/0.373				

### 4.3 Limitations

This study faced several limitations. First, despite prior studies on dementia interventions, this study only established a joint intervention group for MCI due to budget and staffing constraints, omitting a separate control group for analysis. Second, the sample’s representativeness is limited as participants were from urban areas with certain economic levels; future research should broaden inclusion criteria, such as patients from surrounding cities and patients with different economic and educational levels, to enhance sample diversity. Third, the absence of blinding for subjects and interveners, with only data analysts being blind, might bias behaviors, performances, and subjective responses. Fourth, this study was compared with a blank control group, and the results showed that the cognitive function of patients was improved, but it was not compared with other cognitive training interventions. Future studies can focus on comparing the combination of CCT and OT with other activity groups (such as exercise and multi-component intervention). Lastly, the study’s 3-month follow-up may not suffice to ascertain CCT and OT’s long-term effects, despite significant results. Future research should include longer interventions and follow-ups.

## 5 Conclusion

The results of this research reveal that combining CCT with OT for people with MCI can help improve their cognitive function, enhance their independence in compliance with continuing nursing care, and maintain their anxiety and self-care ability at a stable level. Future studies should explore the effects of CCT and OT separately and in combination on each cognitive domain and deeply explore the experiences, feelings, promoting factors, and obstacles to CCT and OT of people living with MCI.

## Data availability statement

The original contributions presented in the study are included in the article/Supplementary material, further inquiries can be directed to the corresponding author.

## Ethics statement

This study has been approved by the Ethics Committee of the Nursing School of Jilin University (approval number: 2022062201) and implemented by the Declaration of Helsinki. All participants were fully informed about the voluntary nature of their participation and their right to withdraw from the study at any time. Data collection takes place only with the participants' written informed consent, and all study data is held using a secure and encrypted platform.

## Author contributions

XW: Conceptualization, Data curation, Investigation, Methodology, Writing – original draft, Writing – review & editing. SS: Conceptualization, Data curation, Investigation, Methodology, Writing – original draft, Writing – review & editing. HT: Data curation, Writing – review & editing. HC: Data curation, Writing – review & editing. LZ: Writing – review & editing, Conceptualization, Formal analysis. YS: Writing – review & editing, Conceptualization, Formal analysis. ML: Writing – review & editing. YW: Supervision, Writing – review & editing, Conceptualization, Validation.

## Footnotes

^1^ www.random.org
